# Minimal Surviving Inoculum in Collective Antibiotic Resistance

**DOI:** 10.1128/mbio.02456-22

**Published:** 2023-04-06

**Authors:** Lukas Geyrhofer, Philip Ruelens, Andrew D. Farr, Diego Pesce, J. Arjan G. M. de Visser, Naama Brenner

**Affiliations:** a Technion–Israel Institute of Technology, Haifa, Israel; b Wageningen University & Research, Wageningen, Netherlands; c University of Leuven, Leuven, Belgium; d Max Planck Institute for Evolutionary Biology, Plön, Germany; Eidgenossische Technische Hochschule Zurich; Emory University

**Keywords:** antibiotic resistance, beta-lactamase, inoculum effect, mathematical modeling, bacterial collective dynamics, population dynamics

## Abstract

A common strategy used by bacteria to resist antibiotics is enzymatic degradation or modification. This reduces the antibiotic threat in the environment and is therefore potentially a collective mechanism that also enhances the survival of nearby cells. Collective resistance is of clinical significance, yet a quantitative understanding at the population level is still incomplete. Here, we develop a general theoretical framework of collective resistance by antibiotic degradation. Our modeling study reveals that population survival crucially depends on the ratio of timescales of two processes: the rates of population death and antibiotic removal. However, it is insensitive to molecular, biological, and kinetic details of the underlying processes that give rise to these timescales. Another important aspect of antibiotic degradation is the degree of cooperativity, related to the permeability of the cell wall to antibiotics and enzymes. These observations motivate a coarse-grained, phenomenological model, with two compound parameters representing the population’s race to survival and single-cell effective resistance. We propose a simple experimental assay to measure the dose-dependent minimal surviving inoculum and apply it to Escherichia coli expressing several types of β-lactamase. Experimental data analyzed within the theoretical framework corroborate it with good agreement. Our simple model may serve as a reference for more complex situations, such as heterogeneous bacterial communities.

## INTRODUCTION

Antibiotic resistance is an outstanding global health problem (1, 2). Much research has been devoted to understanding the molecular mechanisms utilized by bacteria to resist antibiotics, and multiple resistance and tolerance mechanisms have been discovered and described in the past decades (3). It is increasingly apparent that antibiotic resistance also depends on population-level effects, which can be broadly categorized into two classes. In the first class, the resistance of one cell positively affects the survival of other cells nearby. This collective (or cooperative) resistance involves decreasing the effective concentration of antibiotics in the environment, for example, via binding to cellular components or by enzymatic degradation (4, 5). Such a decrease positively affects all bacteria in the shared environment. In the second class, efforts of an individual cell to resist antibiotics might harm its neighboring cells, for example, when efflux pumps keep the internal antibiotic concentration low at the expense of its local increases in the surrounding environment (6).

In this work, we concentrate on the first, collective form of antibiotic resistance. In particular, we focus on the production of enzymes that degrade or modify antibiotics, rendering them ineffective. In some cases, these enzymes are secreted outside the cells (7, 8), while in others, most of the degradation takes place inside the cell (9). β-Lactamases are the best-known enzymes implementing such a degradation strategy by hydrolyzing β-lactam antibiotics, both inside the periplasm and leaking out of the cell through outer membrane porins (8, 10), but several other examples are also known (11, 12). The result is a gradual removal of antibiotic in the environment, which potentially alleviates stress and aids the survival of nearby cells. This helps resistant cells to survive and establish a population (13, 14). Perhaps more conspicuously, this strategy may also enhance the survival of nearby sensitive (nonresistant) cells: once the antibiotic concentration is reduced below their threshold for growth (9, 15), the sensitive population can expand and even compete for resources with the resistant cells (14, 16–19).

In collective resistance mechanisms, the size of the population matters: first, the time-window available for action before extinction depends directly on population size. In addition, more cells produce more degrading enzyme, relieving antimicrobial stress faster and enhancing the probability of recovery before population extinction. Thus, collective resistance exhibits an inoculum effect (20, 21). This makes the standard measure of a MIC, defined as the minimal antibiotic concentration that prevents bacterial growth after 20 h using an inoculum density of $5\times 10^{5}$ cells/mL (22, 23), sensitive to variation in initial conditions. Previous work has addressed this inoculum effect with several different approaches. Artemova et al. (24) studied selection for resistance and found that the single-cell MIC (scMIC), defined for an isolated cell, is the primary determinant of fitness of a resistant strain. More recently several models were tested to describe measurements of inoculum-dependent MIC in different cases (25). Saebelfeld et al. (14) used a simple branching model to predict the MIC of resistant strains in the absence of social interactions, as a reference to detect collective resistance. In addition, the model investigated by Abel Zur Wiesch et al. (26), based purely on binding kinetics of antibiotics to its target, resulted in the emergent phenomena of an inoculum effect and an explanation for persister cells within populations.

Here, we are interested in the population dynamics of collective antibiotic resistance and highlight a range between cooperative and selfish aspects of such resistance. Intuitively, it may seem that enzymes hydrolyzing antibiotics tend to be public if they are excreted and more private if degradation happens inside the cell. However, intracellular degradation reduces the external antibiotic concentration and therefore has some effect also on other cells. Our modeling framework allows for quantifying a varying level of privatization in collective resistance. We describe the sensitivity of a population to antibiotics by an inoculum-dependent MIC, or alternatively, a minimal surviving inoculum (MSI) that can overcome a given antibiotic concentration. Using mathematical modeling, we show that the shape of this dose-dependent MSI curve has universal features and is only weakly dependent on molecular details and reaction kinetics. Rather, it reflects a relationship between the timescales of the population death rate, the antibiotic removal rate, and the level of privatization of the antibiotic-removal mechanism. We propose a simple experimental assay to determine the dose-dependent MSI curve and show that our predictions are in agreement with experimental data.

## RESULTS

### Dynamic model for collective race to survival.

In a bacterial population with no constraints, the number of cells *N*(*t*) grows exponentially in time *t* with a growth rate α:

(1) $$\dot{N}=\alpha N\Rightarrow N(t)=N_{0}e^{\alpha t}.$$

**FIG 1:**
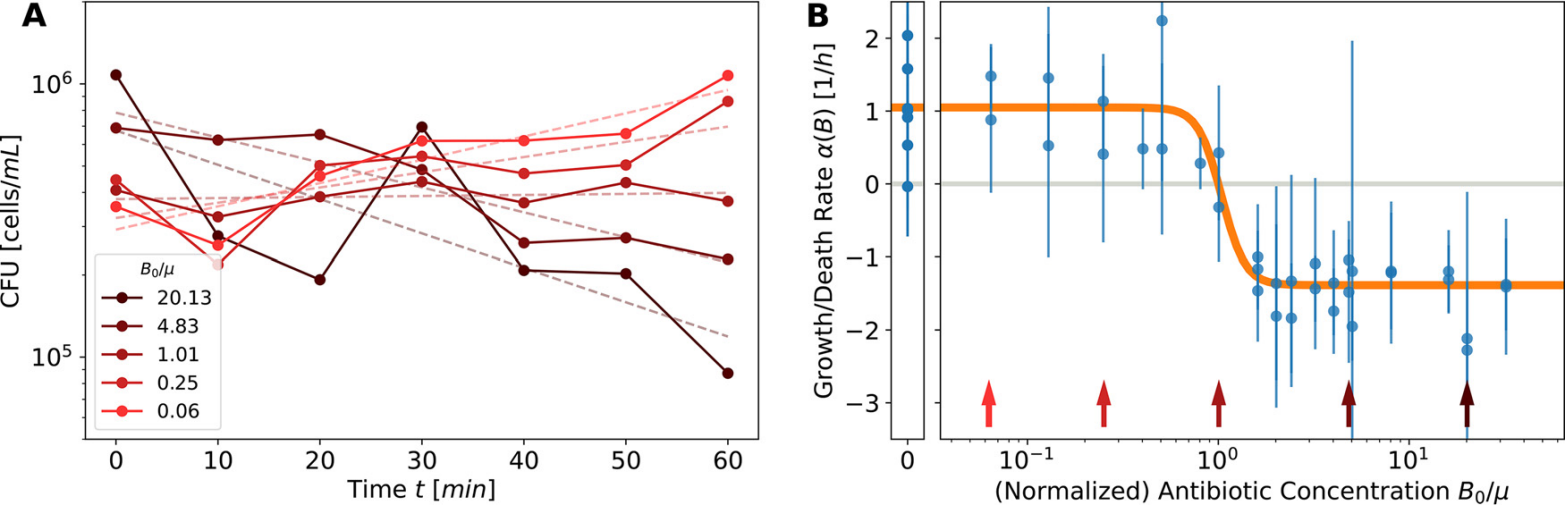
Effect of cefotaxime (CTX) on growth of susceptible E. coli populations using conventional kill curves. (A) Averaged trajectories from 2 replicates of CFU counts at various antibiotic concentrations, specified in normalized units (see panel B). Dashed lines indicate the estimated rate of growth/death over 1 h; colors correspond to arrows in the right panel. (B) Growth rate α(*B*) plotted as a function of CTX concentration *B*/μ, normalized such that at the turning point of zero growth the concentration is *B*/μ = 1. In the absence of CTX, (*B*_0_/μ = 0; left narrow panel), bacteria grow with a rate α_0_, which depends on environmental conditions. Around the threshold of *B*/μ ≈ 1, the growth rate decreases sharply and then saturates at a negative rate –γα_0_. Each data point in panel B is extracted as the logarithmic slope, a few examples of which are shown in panel A. This estimation is labor- and time-intensive, and can contain large errors. The method described in this manuscript circumvents some of these problems. Note that for the susceptible strains used here, the antibiotic concentration is not expected to change, in contrast to the resistant strains discussed in the remainder of the manuscript.

The effect of antibiotics on a population can be described by a dependence of its growth rate on antibiotic concentration *B*. Figure 1A shows this dependence, α(*B*), measured for susceptible Escherichia coli cells exposed to different concentrations of cefotaxime (CTX), a cephalosporin-class β-lactam. In the absence of antibiotics (*B* = 0), the population growth rate α_0_ reflects the physiology of the specific bacterial strain and the nature of the environment, e.g., medium composition. An increasing antibiotic concentration reduces this growth rate sharply around a threshold concentration, *B* ≈ μ. Above this threshold, growth rate α becomes negative as cells are killed and the population size decreases. Further increasing the antibiotic concentration, it has been observed that the rate of death levels out to a constant rate proportional to the growth rate in the absence of antibiotics (27, 28).

A good mathematical description of this dependence is a decreasing sigmoidal curve (a Hill function) (29),

(2) $$\alpha (B)=\alpha_{0}\;\frac{1\;-\;{\left(B/\mu \right)}^{\kappa }}{1\;+\;{\left(B/\mu \right)}^{\kappa }/\gamma }.$$

Here, μ is the concentration at which growth decreases to zero and turns into death (introduced in reference 29 as “pharmacodynamic MIC”), κ is the steepness of the decrease around this threshold, and –γα_0_ is the maximal death rate. This formulation helps to separate the dependence of growth on medium or strain, through α_0_, from the pharmacodynamics of the antibiotics described by the sigmoidal function (27). (For equivalence to previous formulations, see Text S1 in the supplemental material).

10.1128/mbio.02456-22.1 TEXT S1 AppendixText S1, PDF file, 0.9 MB. Copyright © 2023 Geyrhofer et al. 2023 Geyrhofer et al. https://creativecommons.org/licenses/by/4.0/ This content is distributed under the terms of the Creative Commons Attribution 4.0 International license.

Consider a finite population placed in a closed environment with growth medium and an initial concentration of antibiotics *B*_0_ larger than the threshold μ. As a result of this antibiotic exposure, the population size of cells begins to decrease as some cells die. Meanwhile, surviving cells produce enzymes that break down or inactivate the antibiotics, lowering the antibiotic concentration. These two processes define a dynamic race to survival in which antibiotics must be reduced below a threshold before all cells have been killed. The success of this resistance strategy therefore depends on the relative rates of these two processes but also on the initial population size and initial amount of antibiotics. A larger initial population extends the time window to achieve this goal, while more antibiotics decrease the time for the population to degrade antibiotics before it is extinguished.

Chemical reactions that reduce the antibiotic concentration *B* can be implemented in different strains and conditions by various kinetic processes. To illustrate the race to survival, we consider the production of an antibiotic-degrading enzyme *E* by each cell with a rate ρ. In turn, this enzyme degrades antibiotics *B* in a first-order biochemical reaction with catalytic efficiency ϵ:

(3a) $$\dot{N}=\alpha (B)N,$$

(3b) $$\dot{E}=\rho N,$$

(3c) $$\dot{B}=-\epsilon \mathrm{EB}.$$

**FIG 2:**
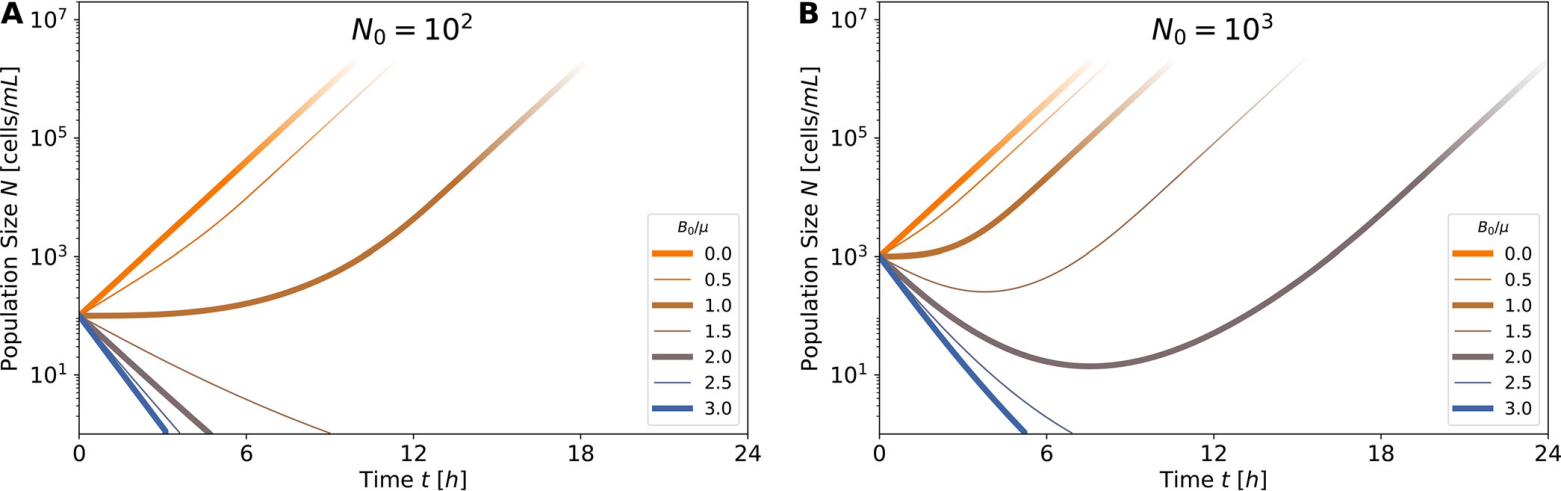
Survival depends on inoculum size and antibiotic concentration. (A and B) Population size across time, *N*(*t*), computed numerically from the model (equation 3), with various parameters: inoculum size of *N*_0_ = 100 (A) and *N*_0_ = 1,000 (B); relative initial antibiotic concentration *B*_0_/μ between 0 and 3 (line colors; see legend). For *B*_0_/μ > 1, the bacterial population initially decays but may recover depending on inoculum size. For example, at antibiotic concentrations of *B*_0_/μ = 2 (purple line), the larger inoculum (B) recovers and survives, while the smaller one (A) does not. Extinction is escaped if enough enzyme is produced during the initial population decay, such that antibiotics are reduced below the threshold concentration μ rapidly enough. In all simulations, ρϵ = 10^−3^.

With the model defined in equation 3a to c, we can integrate *N*(*t*), *B*(*t*), and *E*(*t*) over time. Examples of numerically obtained trajectories are depicted in Fig. 2 for *N*(*t*), with various initial antibiotic concentrations (colors) and inoculum sizes (panels). (An extension of this figure can be found in Fig. S1.5 in Text S1.) The most crucial property determining the population’s fate is whether or not it drops below a single cell, *N*(*t*) < 1, indicating its extinction. This is the lower limit shown in the figure; if any trajectory decreases below this limit, the population is considered extinct. Note that we define extinction at a single cell but neglect stochastic effects arising from discreteness of the population. It can be seen that for small antibiotic concentrations (orange and light brown curves), the population increases exponentially and will continue to do so until it reaches saturation (not modeled here). In contrast, for larger antibiotic concentrations (darker purple to blue curves) it eventually drops below one cell and is considered extinct. Interestingly, for larger inoculum size, at intermediate antibiotic concentrations the population starts to decrease, but as antibiotic is degraded, it then turns around and succeeds to grow. Examples of this behavior can be found in Fig. 2.

### Minimal surviving inoculum.

The standard measure of MIC is used to characterize a threshold of initial antibiotic concentration *B*_0_ beyond which there is no growth at long times, with a standardized inoculum and timescale of observation (30). However, our model indicates that collective resistance is dynamic and is determined by the interplay of two processes: cell death and antibiotic degradation. This race to survival implies an inoculum effect: MIC depends on inoculum size (20, 25). Turning this dependence around, we may think of the minimal surviving inoculum as a function of antibiotic concentration: this minimal surviving inoculum (MSI) is a curve, *N*_0_(*B*_0_), rather than a single quantity such as MIC.

Using the model in equation 3a to c, we can develop an approximation to estimate this MSI curve. If the initial antibiotic concentration is large enough, most of the race-to-survival dynamics takes place with an approximately constant death rate. Thus, we approximate the sigmoid function for large antibiotic concentrations, *B* ≫ μ, with

(4) $$\alpha (B)\approx -\alpha_{0}\gamma \;.$$

This approximation allows us to find solutions to our model, which in turn provide an expression of the MSI curve, *N*_0_(*B*_0_) (see Text S1, section S1.1 for derivation):

(5) $$N_{0}\;\geq \;\tau \;\log(B_{0}/\mu ).$$

**FIG 3:**
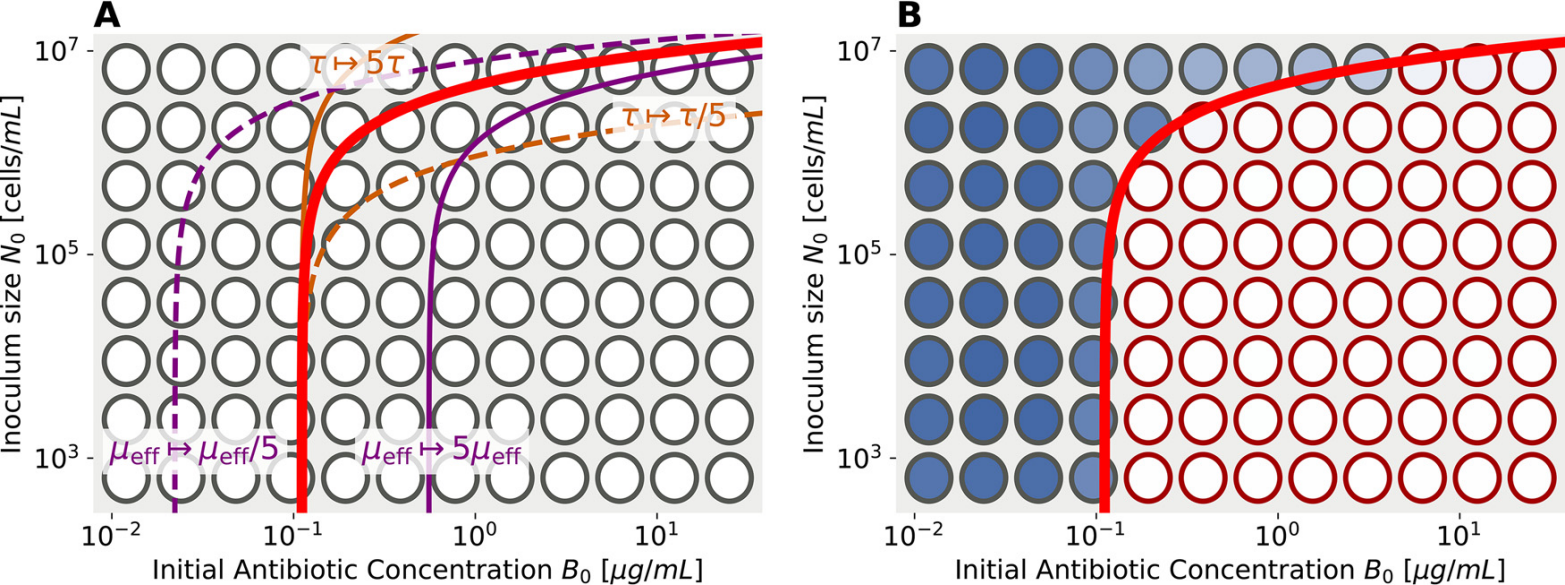
The universal MSI curve: parameters and experiment. (A) The shape of the MSI curve (equation 5), plotted in logarithmic axes (red curve). This curve only depends on its two parameters τ and μ_eff_. Increasing τ (brown curves) stretches or compresses the MSI curve in the direction of inoculum size (*y* axis). Changes in the parameter μ_eff_ (purple curves) induce a shift of the MSI curve in the direction of antibiotic concentration (*x* axis), which includes shifts from the inherent ability to withstand antibiotics, but also the effects of privatization described in section II C “Single-cell privatization of resistance.” (B) Experimental measurement of the MSI curve with data from a resistant E. coli strain. A microwell plate is started with serial dilutions of the inoculum size *N*_0_ and antibiotic concentrations *B*_0_ along the two axes. The threshold for survival is apparent by color after a long time of growth: blue wells have a population in them, while white cells do not. If the ranges of dilutions are chosen appropriately, the MSI curve (red line) appears with its typical universal shape as the boundary between wells with surviving and extinct populations. Protocol details are described in Materials and Methods.

The MSI curve, as shown in Fig. 3 (red lines), is an increasing function: as the initial antibiotic concentration increases, a larger inoculum is needed to ensure survival. Its simple form is largely robust with respect to the details of the mechanism for antibiotic degradation or inactivation (see Text S1, section S1.1).

Let us consider the interpretation of the two parameters determining the MSI curve. The first parameter, μ is the threshold antibiotic concentration allowing growth (see Fig. 1), which sets the scale for antibiotic concentration. In terms of the plots presented in Fig. 3A, μ is the intercept of the MSI curve at the edge at which antibiotic concentration varies, corresponding to the small-inoculum limit (which approximates the single-cell MIC [24] or pharmacodynamic MIC [29]).

In addition, the MSI depends on the parameter

(6) $$\tau =\frac{{\left(\alpha_{0}\gamma \right)}^{2}}{\epsilon \rho },$$

representing a ratio of timescale between the death rate, α_0_γ, and the two rates involved in antibiotic degradation, ϵ and ρ. As the degradation process consists of two rates here, the death rate—against which they are compared—appears squared. (Details on derivation are presented in Text S1.) Large τ corresponds to fast killing of bacteria relative to degradation and thus results in a higher MSI. In terms of the plot of Fig. 3, the parameter τ multiplies the curve by a constant, stretching it along the population size axis (see Fig. 3A). Note that when comparing to data, population size is measured in cells/mL, and τ has the same units; we assume that the experimental volume remains fixed, so numbers and concentrations are related in a straightforward manner.

If the initial antibiotic concentration is not very high, but is still above threshold, we can employ a different approximation for the growth rate. In Text S1, section S1.1.2, we show that $\alpha (B)\approx -\alpha_{0}\frac{\kappa \gamma }{1\;+\;\gamma }\log(B_{}/\mu )$, which is valid close to *B* ≈ μ, still allows us to find solutions to our model. In general, a correction close to *B* ≈ μ should not affect the shape of the MSI curve too much, as the asymptotic behavior for large *B*_0_ is still given by equation 5.

Importantly, although we developed equation 5 for a specific model of enzyme degradation, the shape of the MSI curve turns out to be insensitive to many details of the kinetics of collective resistance, as we show in Text S1, section S1.1, for spontaneous degradation, irreversible binding of antibiotics to fragments of dead cells, and absorption and internal degradation. In the different mechanisms we analyzed, the extant parameter τ depends on combinations of underlying molecular parameters for different kinetic mechanisms. Nevertheless, its interpretation is always the same: τ represents the ratio between the rate of population death α_0_γ and the rate of reduction in antibiotic concentration. This suggests a coarse-grained approach that utilizes the MSI curve with its two parameters, μ and τ, as the basic empirical observation.

### Single-cell privatization of resistance.

**FIG 4:**
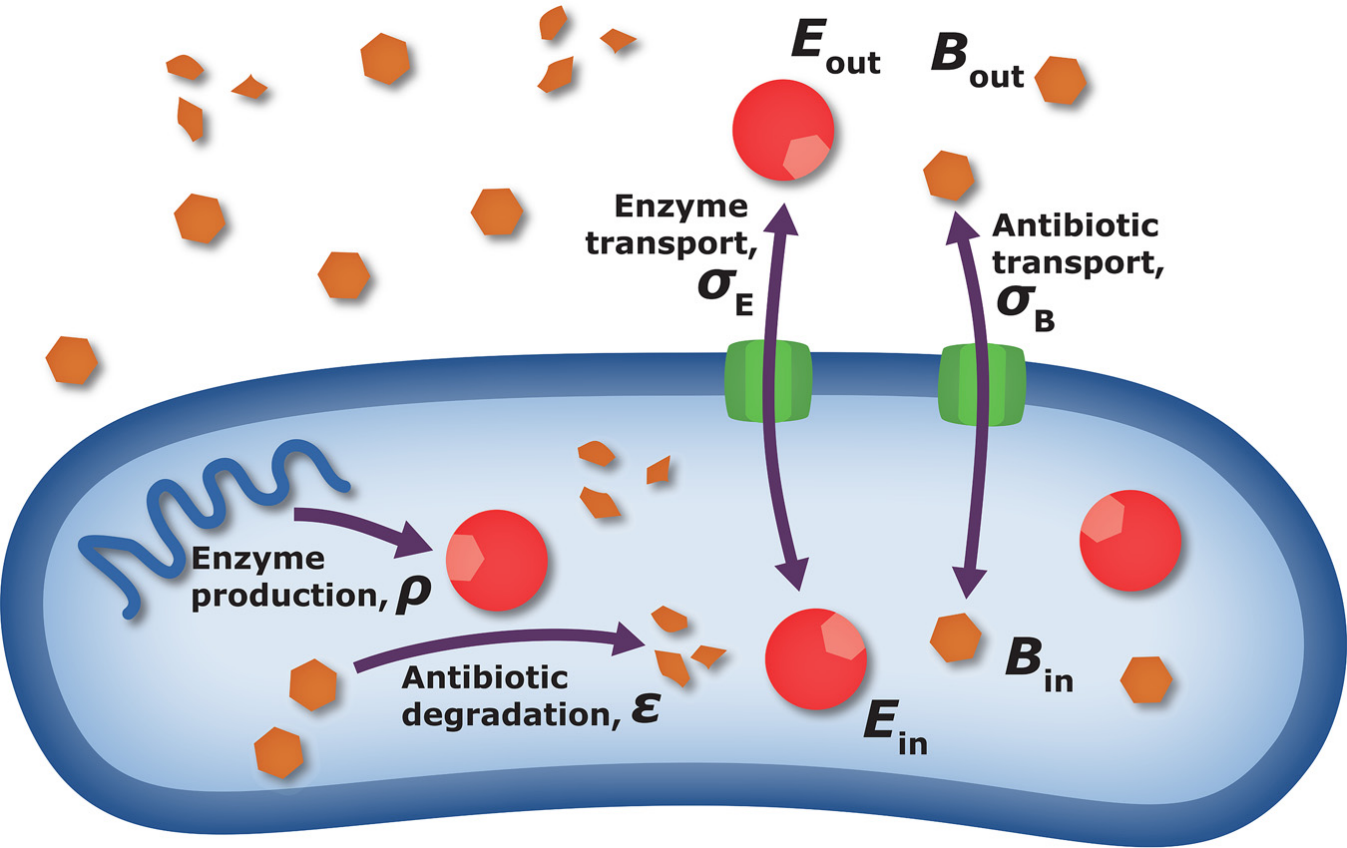
Factors affecting privatization of resistance. To describe different levels of privatization/cooperativity in antibiotic resistance, we include in our model external concentrations of both enzyme and antibiotics. The transport between external and internal spaces is governed by permeabilities σ_E_, σ_B_. Enzyme production and antibiotic catalytic efficiency are described by ρ and ϵ respectively. The level of privatization is determined by the relative importance and speed of these processes.

Until now, we have assumed that resistance is completely cooperative; produced enzymes are secreted to the common environment and directly affect the global antibiotic concentration. In this view, the antibiotic concentration is homogeneous across space and, hence, the same for the cell which produces the resistant enzymes and any other nearby cells which benefit from this production. In reality, at least part of the degradation takes place inside the cell (often in the periplasmic space), thus providing an increased benefit to the producing cell relative to its neighbors. In environments which are not mixed rapidly enough, even if enzymes are excreted, they are more abundant in the vicinity of producing cells and will take time to diffuse away, again providing an increased benefit to the secreting cell. Our goal is now to quantify the level of privatization (or, inversely, of cooperation) in collective antibiotic resistance. A full model should include transport and diffusion of concentrations in space. As a first approximation, we include the leading distinction between internal and external concentrations of both enzyme and antibiotics: *E*_in_, *E*_out_, *B*_in_, *B*_out_ (Fig. 4). The coupled kinetic equations for these variables can be found in Text S1, section S1.2. We assume that concentrations inside the cell equilibrate much faster than outside, corresponding to its volume being tiny compared to the practically infinite reservoir of the external environment. This assumption allows us to estimate the internal concentrations as dynamically dependent on the outer concentrations,

(7a) $$E_{\text{in}}\approx E_{\text{out}}\;+\frac{\rho }{\sigma_{\text{E}}},$$

(7b) $$B_{\text{in}}\approx {\left(1\;+\frac{\epsilon \rho }{\sigma_{\text{E}}\sigma_{\text{B}}}\right)}^{-1}B_{\text{out}}={\left(1\;+\;\Phi \right)}^{-1}B_{\text{out}}.$$

Here, σ_E_ and σ_B_ are permeabilities for enzymes and antibiotics, respectively, indicating the rate of them passing through the cell wall in either direction. High permeability will prevent the buildup of a concentration difference across the cell wall and thus will promote cooperativity, while low permeability will promote privatization.

The effect of transport on enzyme concentration is seen in equation 7a, which describes the difference between internal and external concentrations: internal is always larger, and high-production ρ or, alternatively, low-permeability σ_E_ work to increase this difference. Usually, external enzyme concentrations are smaller than internal concentrations, which can be seen directly from equation 7a.

Antibiotic concentration, in contrast, is always lower inside the cell (see equation 7b). The outer concentration, *B*_out_, is multiplied by a dimensionless factor, (1 = Φ)^−1^, that depends on both production and transport parameters: intuitively, antibiotics are supplied on the outside and first need to enter the cell, while being reduced. This reduction is faster inside cells than outside, as the enzyme concentration is larger inside. Overall, high production and low permeability support a larger antibiotic concentration difference, as is reflected in equation 7b.

We refer to the compound parameter appearing in equation 7b as a “privatization parameter” Φ = ϵρ/σ_E_σ_B_. High privatization occurs for Φ ≫ 1, corresponding to very high enzyme production, ρ, high catalytic efficiency, ϵ, or low transport coefficients. In this regime, cells degrade the antibiotics mostly privately by lowering their internal antibiotic concentration and not sharing degrading enzymes with neighbors. At the other end, Φ ≪ 1, antibiotic and enzyme concentrations are almost equal inside and outside the cell, and inactivation of antibiotic occurs in the public domain of the shared environment, resulting in maximal cooperativity to all cells. In this limit we effectively return to the first, naive model, where no distinction was made between internal and external concentrations. The privatization parameter Φ enables us to interpolate between these two extremes. It is a compound parameter; the level of privatization is determined by the difference in concentration between the intracellular and extracellular environment, resulting from catalytic efficiency, production, transport, or possibly other microscopic processes.

In a real experimental setting, it is difficult—or even impossible—to measure internal concentrations of drug and enzyme, and usually only external concentrations are measurable. Yet, it is the internal concentration which directly affects how antibiotics alter the growth of a cell or cause its death. As can be seen from equation 2, the effect of antibiotics on growth rate is measured in units of μ and appears as “normalized concentration” *B*/μ in all instances. Thus, equation 7b allows us to translate between the two concentrations, *B*_in_ and *B*_out_, and we can write the effective growth rate as a function of the observable external antibiotic concentration:

(8) $$\alpha \left(\frac{B_{\text{in}}}{\mu }\right)=\alpha \left(\frac{B_{\text{out}}}{\left(1\;+\;\Phi \right)\mu }\right)=\alpha (\frac{B_{\text{out}}}{\mu_{\text{eff}}}).$$

The discrepancy between (unmeasurable) internal and (measurable) external concentrations can be attributed to a modification of the growth threshold μ, which we define as

(9) $$\mu_{\text{eff}}=\left(1\;+\;\Phi \right)\mu .$$

With this result, we need to modify the MSI curve obtained in the previous section, which can now be stated in terms of the quantity, *B*_0_ = *B*_out_(*t* = 0), by exchanging the previous μ with μ_eff_:

(10) $$N_{0}\;\geq \;\tau \;\log(B_{0}/\mu_{\text{eff}}).$$

This MSI curve in equation 10 has the same functional form as before with two fitting parameters, τ and μ_eff_, but now one of them also includes transport properties; it is an effective parameter that deals with the difference between external and internal concentrations. Specifically, if privatization increases (smaller permeabilities), then μ_eff_ will also increase, and vice versa. Thus, the effects of this extension for our model are already contained in Fig. 3A, as we only infer μ_eff_ from experimental data.

The two parameters in the MSI curve are, in fact, not independent of one another. We have encountered τ as a ratio between the timescales of cell death α_0_γ and antibiotic degradation (Text S1 deals with different processes of how antibiotic concentration decreases). The transport processes for both antibiotics and enzyme generate a third timescale in our model. Thus, we may write the privatization parameter as

(11) $$\Phi =\frac{\epsilon \rho }{\sigma_{\text{E}}\sigma_{\text{B}}}=\frac{\epsilon \rho }{{\left(\alpha_{0}\gamma \right)}^{2}}\;\frac{{\left(\alpha_{0}\gamma \right)}^{2}}{\sigma_{\text{E}}\sigma_{\text{B}}}=\frac{\varphi }{\tau }.$$

This representation is useful, because it separates the race between single-cell resistance and antibiotics, represented by τ, from the relative extent of cooperativity. Using equation 11, we can predict a relation between the two fitting parameters of the MSI, τ and μ_eff_:

(12) $$\mu_{\text{eff}}=\left(1\;+\frac{\varphi }{\tau }\right)\mu .$$

While each experiment will result in an MSI curve with different parameters, they are not expected to be randomly scattered in the plane (μ_eff_, τ), but rather to be located on a curve defined by equation 12. To test this prediction, we present an experimental assay to measure the MSI curve below.

### Experimental validations.

An experimental procedure that measures the antibiotic dose-dependent MSI curve is conceptually straightforward to do with a 96-well assay. Populations are inoculated with increasing inoculum size *N*_0_ along one axis and with increasing antibiotic concentrations *B*_0_ along the other (Fig. 3). Concentrations along the axes are chosen appropriately, such that the shape of the MSI curve fits on the plate (which requires some prior knowledge about the ranges of the MSI curve). Following overnight incubation of the cultures, a clear difference is visible between surviving and nonsurviving populations. As an example, Fig. 3B shows all optical density (OD) measurements of wells in different shades of blue; white indicates no growth in the well. Subsequently, one may fit equation 10 with its two parameters, τ and μ_eff_, to this transition from surviving to nonsurviving populations on the plate. In Fig. 3B it is depicted by a red line. All steps in our algorithm to extract parameters from OD measurements of plates are described in detail in Text S1, section S1.3. The first parameter, τ, multiplies the MSI curve and mostly determines the intersection on the *N*_0_ edge of the plate for large antibiotic concentrations. The second parameter, μ_eff_, corresponds to the intersection of the MSI curve on the *B*_0_ edge of the plate for small initial population sizes, which approximates the single-cell MIC (24).

We applied this experimental procedure to several E. coli strains expressing various β-lactamases. In a first set of experiments, we used mutants of E. coli MG1655 with a chromosomally integrated gene encoding and expressing β-lactamase TEM-1 at a single intermediate level. These include a susceptible wild type without the TEM-1 gene (suscWT) and a consecutive series of mutations in the TEM-1 enzyme (TEM1, G238S, E104K/G238S, E104K/M182T/G238S), which exhibit increasing catalytic efficiency ϵ for cefotaxime (31). A second set of experiments used E. coli BW27783, where two of these TEM alleles (G238S and E104K/M182T/G238S) are located on plasmid pBAD322T, and their expression is controllable using an arabinose-inducible promoter. This allows us to manipulate enzyme production ρ; the subset of mutations in TEM-1 again alters the catalytic efficiency ϵ to moderate and high values. A detailed experimental protocol is described in Materials and Methods.

For most experiments, the MSI curve can be fit nicely to the data and the effective parameter estimates inferred. An example of such a fit is shown in Fig. 3B; all other data are presented in Fig. S1.3 and S1.4 in Text S1. In some cases, the low resolution of a 96-well plate caused a large uncertainty in parameter values. Nevertheless, our repeated experiments yielded similar parameter values in practically all tested cases.

**FIG 5:**
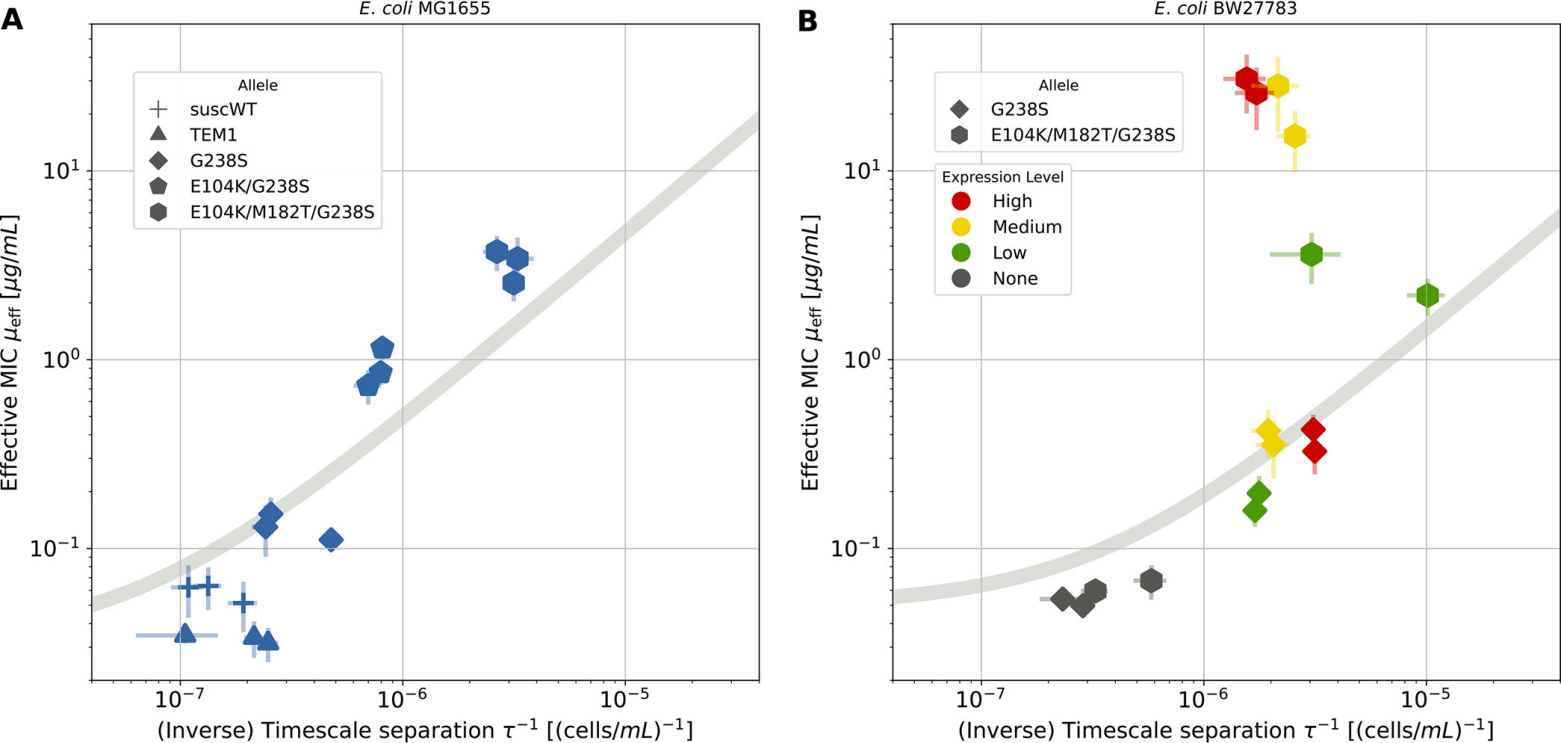
Relation between μ_eff_ and τ inferred from measured MSI curves using β-lactamase-producing strains with various resistance levels. (A) Best fit of equation 9 for strains expressing TEM-1 variants with different catalytic efficiency ε toward cefotaxime. We also included a susceptible strain (suscWT) without TEM-1. (B) Best fit of equation 9 for a strain with low (G238S; rhombus) or high catalytic efficiency ϵ (E104K/M182T/G238S; hexagon) and different expression levels ρ. The gray line corresponds to equation 12, with φ fitted from the data. Error bars of individual data points are estimated in the fitting algorithm (see Text S1, section S1.3). Each symbol corresponds to a single MSI experiment; overall, the same conditions were applied in duplicate (B) or triplicate (A).

In our experimental setup only the gene (including its promoter) for TEM-1 is changed within both sets of experiments. Thus, we can expect that the transport properties, which have been separated out and captured by the parameter φ in equation 12, should be unchanged. Under this assumption, equation 12 predicts a relation between the two fitting parameters, τ and μ_eff_, across different plates (gray lines in Fig. 5).

The resulting fitting parameters, as estimated for our collection of experiments, are shown in Fig. 5. Panel A shows the results of our first set of experiments with a strain without TEM-1 and strains expressing TEM-1 and its three mutants at a fixed expression level. The data points are not scattered in the plane, and they follow the predicted curve to a good extent with TEM-1, the single, double, and triple mutant being positioned increasingly higher along the curve, mirroring their respective increase in resistance level (31). Panel B presents the results for the second set of experiments exhibiting different expression levels of two TEM variants (single and triple mutant). While the single mutant follows our prediction, the triple mutant deviates from the curve at medium and high expression levels. We speculate that this deviation could arise due to heavy stress on the bacteria from the overexpression of β-lactamase, which significantly lowers their overall growth rate α_0_ (Fig. S1.6). Moreover, it is possible that the external enzyme concentration is not negligible and that the approximation equation 7b is invalid. In these cases, the relation between the two fitting parameters is not expected to obey the scaling relation. However, in general, the compound parameter estimates for the different experiments clearly do not scatter randomly in the parameter plane but are strongly correlated with one another, and—except for the two outlying conditions—are well described by our model.

## DISCUSSION

Collective resistance is a phenomenon by which a bacterial population can survive an antibiotic dose which a single bacterium cannot (18). This collective behavior may have a profound influence on the effectiveness of an antibiotic therapy and thus may pose serious health risks. Using mathematical modeling, we studied collective resistance via the common mechanism of antibiotic degradation or modification and proposed a unifying framework to describe the minimal population size needed to survive a specific antibiotic concentration. We developed a conveniently simple approximation of our model that allows us to determine the dose-dependent minimal surviving inoculum (MSI) curve. This curve is a generally increasing function, with a larger inoculum being able to survive increasingly higher antibiotic concentrations. Interestingly, we showed that basic features of this curve, and by extension of collective resistance via antibiotic degradation or modification, are insensitive to details of the exact resistance mechanism. Rather than kinetic parameters, the central quantities determining survival are ratios between timescales, when populations race against time to survive by collectively decreasing the antibiotic concentration.

A first parameter central to the MSI curve describes the ratio of timescales between population extinction by antibiotic killing and the competing process of antibiotic degradation. This parameter, referred to as τ in our model, is agnostic to the exact mechanism by which the antibiotic is degraded or modified (Text S1). This is in line with previous work (32), which also reported that it is hard or even impossible to determine details of the degradation mechanism by only observing microbial population dynamics. The hiding of microscopic kinetic details from higher-population-level dynamics is a form of buffering between levels of organization. While insights into the molecular mechanisms need to be known for a targeted antimicrobial therapy to be effective, the relative robustness of population-level dynamics, as shown by our modeling, is important for a basic understanding of resistance at the population or community level.

The second central feature in our model is the level of privatization of the resistance mechanism, namely, how much of the degradation or modification of antibiotics takes place in the shared environment, relative to the intracellular environment. Previous work has considered the limit of maximally private degradation which takes place inside the periplasmic space (33). Their relation between internal and external threshold concentration is similar to ours in that limit, consisting of the ratio between hydrolysis rate and permeability. Other works directly incorporated one form of collective resistance, e.g., the lysis of cells as they die and the release of their enzyme content (24). Our general result (equation 9) interpolates between the high privatization limit and the other limit of low privatization, where hydrolysis takes place primarily in the public domain. This provides a coarse-grained phenomenological description that could apply specifically to lysis or secretion.

Although we here studied the MSI curve of single strains, quantifying the level of privatization may have far-reaching consequences for cross-protection between microbial communities and the eco-evolutionary dynamics of antibiotic resistance (14, 34). The relationship between single-cell and population-level MIC was characterized also in previous work, where it was found that the single-cell properties affect long-term evolutionary outcomes (24). Nevertheless, intermediate timescale dynamics of mixed populations can also be strongly affected by the collective dynamics highlighting the importance of the MSI concept. Future work may utilize this concept to characterize the mutual interactions between different strains as they race for survival, potentially enhancing resistance or one another.

Based on our model, we proposed a simple 96-well microtiter-plate assay that allows us to characterize the parameters describing the MSI curve. We provide a detailed protocol in Materials and Methods to perform this assay and extract the relevant parameters, τ and μ_eff_. This method relies on the idea that a 96-well plate can serve to scan inoculum sizes and antibiotic concentrations in parallel and provide a platform for mapping the MSI curve. A similar idea was proposed for characterizing antibiotic persistence, where the exposure time is a critical variable and is varied along one axis of a 96-well plate by changing the time at which the medium is inoculated (35).

The microtiter-plate assay was used to assess the model at two levels. First, we tested the fit of our predicted MSI curve to describe the border between surviving and nonsurviving populations and found good agreement. Second, we performed two sets of experiments, where we independently varied the catalytic efficiency or expression level of an antibiotic-degrading enzyme in bacterial strains. Under the approximation of significant privatization and independence of other physiological properties of the bacteria, we derived a relation between fitting parameters across the set of experiments (equation 12). The results show that this relation agrees rather well for all but two data points from strains expressing a β-lactamase with very high catalytic efficiency (the triple mutant) at high levels (Fig. 5). The current experiments do not allow us to identify the source of this qualitative discrepancy, but since the triple mutant MSI curve was assayed at significantly higher antibiotic concentrations, this may affect cell physiology via the induction of the SOS response and β-lactam-induced filament formation (36) or by a varying level of lysis and release of enzyme. This would modify the privatization parameter, which the model assumes to be fixed along the curve. Moreover, differences in cost of expression between β-lactamase alleles could also lead to differential effects of expression on cell physiology and permeability, potentially affecting the timescale ratios describing the MSI curve.

In summary, our work contributes to identifying generic mechanism-independent parameters that can be inferred from population data. It identifies robust parameter combinations that govern population dynamics in collective resistance. Specifically, it reveals relative timescales in the race for survival of populations which inactivate antibiotics that kill them, as well as levels of cooperation versus privatization of resources in the fight against antibiotics. Our experimental framework adds a dimension to the characterization of antibiotic resistance by a concentration threshold: it extends this notion to an inoculum-dependent threshold relevant for cells utilizing a collective resistance mechanism. Our framework is expected to be amenable for extension also to the interaction between resistant and sensitive strains, which has been partially addressed from a different perspective in previous work (17).

## MATERIALS AND METHODS

### Quantification of bacterial growth with antibiotic dosage.

Methods used to estimate the impact of antibiotic levels on the growth of bacterial cultures—referred to as “kill curves”—were adapted from reference 29. Briefly, Escherichia coli MG1655 galK::SYFP2-FRT was cultured overnight in M9 minimal medium (supplemented with 0.4% glucose, 0.2% Casamino Acids, 1 μg/mL thiamine, 2 μg/mL uracil, and 50 μM IPTG [isopropyl-β-d-thiogalactopyranoside]). Stationary-phase cultures were diluted 1:1,000 into minimal medium and incubated with shaking, and meanwhile, dilution series were made of cefotaxime in minimal medium; 140 μL of each dilution was aliquoted into a row of a 96-well plate. All medium for subsequent culturing was then prewarmed to aid continuous growth of bacteria. After 120 min, the concentration of cells was measured by flow cytometry and diluted in minimal medium to approximately 20 · 10^6^ cells/mL, and 140 μL of cells was added to each well containing cefotaxime. A 20-μL sample was immediately taken, and the plate was incubated with orbital shaking for 60 min at 37°C, with further 20-μL samples taken every 10 min. For each sample a dilution series of 10^−1^ to 10^−3^ was made, and 100 μL of each dilution was plated on minimal medium solidified with agar. Plated cell solution was spread with 12 to 14 3-mm glass beads. Plates were incubated for ~24 h, and CFU were counted from appropriate dilutions.

### Experimentally determining the MSI curve.

The proposed assay exposes different inoculum sizes of bacteria to different concentrations of antibiotics until the boundary between wells with surviving and extinct populations is evident. The population size is varied across the eight rows (A to H) of a 96-well plate, while antibiotic concentrations vary across the 12 columns of the plate. Care should be taken when choosing the antibiotic dilution range to fully capture the MSI curve. The examples shown in this study involved 2-fold dilutions of antibiotic; however, alternative dilution strategies could be used to more appropriately observe the MSI curve.

### Protocol.

1. Grow the strain of interest overnight in a suitable growth medium.
2. Prepare an antibiotic solution in the chosen growth medium that is four times the highest concentration that will be tested.
3. Fill all wells in a sterile 96-well plate with 100 μL of growth medium.
4. Dispense 100 μL of the antibiotic solution with a concentration 4 times the intended final concentration in column 1 of the microtiter plate. Using a multichannel pipette, mix the antibiotics and transfer 100 μL from column 1 to column 2. Mix again and repeat this procedure down to column 12. Discard 100 μL of solution from column 12.
5. Prepare a serial dilution of the bacterial overnight culture in eight appropriate tubes. The highest concentration should be twice the desired highest inoculum. The highest inoculum size in the validation experiments was approximately 1.25 · 10^6^ CFU/mL or 2.5 · 10^5^ CFU/mL. The examples presented in this study involved 4-fold dilutions, which allowed low concentrations of cells (a few hundred cells/mL) to inoculate the final wells in the dilution series, with the primary constraint being the 8 rows of the 96-well plate.
6. Dispense 100 μL of each of the 8 dilutions of cells across each row of the 96-well plate.
7. Incubate the plates at 37°C for 24 h or until satisfactory growth is obtained.
8. Growth can be visually assessed or by spectrophotometric reading (optical density at 600 nm [OD_600_]).

The validation experiments described in this study were performed using the above-mentioned protocol. For the set of experiments using enzyme variants with a different catalytic activity, TEM-1 and three alleles (G238S, E104K/G238S, E104K/M182T/G238S) were amplified from previously described plasmid constructs (37) and introduced into chromosomal *galK* of Escherichia coli MG1655 using the Quick and Easy E. coli gene deletion kit (Gene Bridges). Mutants were selected by selection for ampicillin resistance, and the introduction of β-lactamase genes was confirmed by PCR and Sanger sequencing. The MSI assay was performed with the same minimal medium used for kill-curves. For the set of experiments assessing the effect of expression, TEM-1 alleles G238S and E104K/M182T/G238S were subcloned into pBAD322T behind an arabinose-inducible promoter and transformed in E. coli BW27783 (CGSC no. 12119), which carries a deletion for the arabinose-metabolizing genes (38, 39). Here, the MSI assay was performed in standard LB medium supplemented with 0.1% (high expression), 0.003125% (medium expression) 0.00078125% (low expression), or 0% (no/leaky expression) l-arabinose. For both sets of experiments, growth was measured after 24 h using a Victor3 plate reader (Perkin-Elmer).

### Parameter estimation on plates.

Estimating the two parameters τ and μ_eff_ from the OD of one plate involves multiple steps. First, we estimate a threshold between growth and no growth by applying Otsu’s method to find a threshold value that separates the modes in a bi-modal distribution of OD values. We compute the contour line of this threshold value on each plate to obtain points on a curve close to the growth/no-growth threshold. In the last step we fit the predicted MSI curve, *N*_0_ ≈ τ log(*B*/μ_eff_), to this contour line, which yields values for τ and μ_eff_. The main concepts and equations of all steps are described in more detail in Text S1. The implementation in Python can be found at https://github.com/lukasgeyrhofer/antibiotics.
