# Supplementary material for: Minimal Surviving Inoculum in Collective Antibiotic Resistance
Source: mBio. 2023 Apr 6;14(2):e02456-22. doi: 10.1128/mbio.02456-22 (PMC10128016; doi:10.1128/mbio.02456-22)
Supplement: TEXT S1 [file mbio.02456-22-s0001.pdf]

Appendix S1
Minimal Surviving Inoculum in collective antibiotic resistance

Lukas Geyrhofer[1], Philip Ruelens[2,3], Andrew D. Farr[2,4], Diego Pesce[2], J. Arjan G. M. de Visser[2], and Naama Brenner[1]

[1]Technion – Israel Institute for Technology, Haifa, Israel
[2]Wageningen University & Research, Wageningen, Netherlands
[3]University of Leuven, Leuven, Belgium
[4]Max Planck Institute for Evolutionary Biology, Plön, Germany

S1.1 Universality in antibiotic degradation dynamics

An intriguing theoretical observation is that the MSI curve of Eq. (5) is insensitive to the specific antibiotic decay mechanisms – it is universal to a good approximation. Below, we show that multiple degradation dynamics lead to similar expressions for the curve, utilizing only the two extant parameters τ and μ, that characterize the time scale ratio between cell death and degradation, and antibiotic concentration at zero-growth, respectively. In the main text, we only considered antibiotic degradation by an excreted extracellular enzyme. Other (bio)chemical or biological processes could provide resistance against the action of the antibiotic by reducing its concentration. A few examples of such mechanisms, stated as simple reactions (with rates indicated above arrows), are as follows,

$$\begin{aligned}
\text{Enzymatic degradation} \qquad & N \xrightarrow{\rho} N + E\ , \quad E + B \xrightarrow{\epsilon} E\ , \\
\text{Absorption and Internal degradation} \qquad & N + B \xrightarrow{\zeta} N\ , \\
\text{Irreversible binding to dead cell fragments} \qquad & (N_0 - N) + B \xrightarrow{\nu} \emptyset\ , \\
\text{Spontaneous degradation} \qquad & B \xrightarrow{\chi} \emptyset\ .
\end{aligned} \tag{S1.1}$$

The first reaction in this list corresponds to the dynamics with excreted extracellular enzyme, with linear kinetics, discussed in the main text. In the second reaction, antibiotics is absorbed into the cell, and is degraded by some unspecified cellular reaction inside the cell. In the third example, we consider irreversible binding of antibiotics to fragments of dead cells. Their concentration is proportional to the number of cells that have died during exposure; this expression is relevant as long as population size is decreasing, as these dead cells are assumed to continue binding antibiotics. Finally, we also treat spontaneous environmental degradation of antibiotics, which cells do not actively contribute to.

For all these mechanisms, the dynamics of cells and antibiotics can be described by the dynamical

system,

$$\partial_t N = \alpha(B) N \ , \tag{S1.2a}$$
$$\partial_t B = -f(B, N) \ , \tag{S1.2b}$$

where $f(B, N)$ specifies one of the degradation reactions of antibiotics, as listed above in Eq. (S1.1). Throughout this appendix, we also use the notation ∂_t for derivations with respect to time t, as it helps with the ensuing calculations. Turning the reactions in Eqs. (S1.1) into equations, we find the different mechanisms follow the kinetics:

$$\text{Enzymatic degradation} \qquad \partial_t B = -\epsilon E B \ , \quad \partial_t E = \rho N \ , \tag{S1.3a}$$
$$\text{Absorption and Internal degradation} \qquad \partial_t B = -\zeta B N \ , \tag{S1.3b}$$
$$\text{Irreversible binding to dead cell fragments} \qquad \partial_t B = -\nu (N_0 - N) B \ , \tag{S1.3c}$$
$$\text{Spntaneous environmental degradation} \qquad \partial_t B = -\chi B \ . \tag{S1.3d}$$

Below, we will solve these dynamical systems in detail, leading to a condition on the minimal inoculum size N_0 that can recover from an initial antibiotic concentration B_0:

$$N_0 \gtrsim 1 + \tau \left(\log(B_0/\mu)\right)^{\xi} \ , \quad \xi \sim \mathcal{O}(1) \ . \tag{S1.4}$$

This is a general expression common to all kinetic mechanisms; it defines the universal MSI. Each degradation mechanisms is characterized by a timescale separation τ, which is the ratio between two characteristic rates in that specific mechanism: a population kill rate and an antibiotic degradation rate. The exponent ξ varies slightly depending on the mechanism, but always assumes values close to 1. Moreover, the different approximations for the growth rate $\alpha(B)$ explained in the next paragraph also influence the value of ξ.

In general, the sigmoidal function for growth rate $\alpha(B)$ can not be used in its full form to compute solutions to the dynamics. As observed in Fig. 1, the effective kill-rate is constant for very large antibiotic concentrations. In contrast, for antibiotic concentrations close to $\log\big(B/\mu\big) \approx 0$ (equivalent to $B \approx \mu$), we expand it to linear order in $\log\big(B/\mu\big)$. Mathematically, we will deal with two approximations corresponding to these regimes:

$$\alpha(B) = \alpha_0 \, \frac{1 - (B/\mu)^{\kappa}}{1 + (B/\mu)^{\kappa}/\gamma} \tag{S1.5a}$$
$$\approx \begin{cases} -\alpha_0 \lambda \log\big(B/\mu\big) \ , & B/\mu \sim 1 \ , \\ -\alpha_0 \gamma \ , & B/\mu \gg 1 \end{cases} \tag{S1.5b}$$

with $\lambda = \kappa\gamma/(1+\gamma)$. Both approximations are illustrated in Fig. S1.1. Previous work has mainly considered antibiotic concentrations where the linear regime is negligibly small and the curve can be approximated by a step function [1]. We are here interested also in the tug-of-war dynamics that occurs around the regime of intermediate antibiotic concentrations, where the growth curve is approximately linear. When deriving results below, we will compare these two regimes. Specifically, section S1.1.1 deals with the constant approximation for large enough antibiotic concentrations, while section S1.1.2 treats the logarithmic approximation close to $B_0 \approx \mu$.

In some previous work, the effect of antibiotics on bacterial growth was formalized in $E_{\max}$-models and/or $\Psi_{\max}$-models [2]. There, the effective growth rate α_{eff} is stated as the difference from the maximal

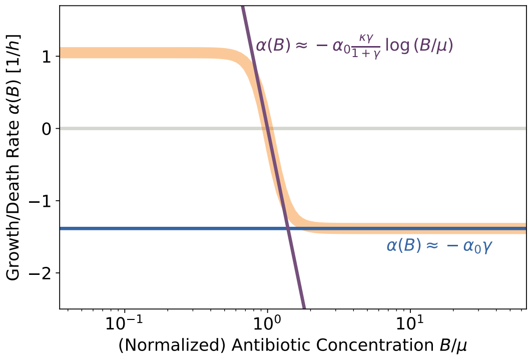

Figure S1.1: **Approximations for effective growth rate.** Two regions are observed, a log-linear regime around MIC and a constant death rate at antibiotic concentrations much larger that MIC.

growth rate $\Psi_{\max} \equiv \alpha_0$,

$$\alpha(B) = \Psi_{\max} - E_{\max} \frac{(B/\mathrm{EC}_{50})^\kappa}{1 + (B/\mathrm{EC}_{50})^\kappa} \tag{S1.6a}$$

$$= \Psi_{\max} - \frac{(\Psi_{\max} - \Psi_{\min})(B/\mathrm{zMIC})^\kappa}{(B/\mathrm{zMIC})^\kappa - \Psi_{\min}/\Psi_{\max}} , \tag{S1.6b}$$

using the notation used in [2]. These parameters are interpreted as follows: $E_{\max}$ is the maximal effect of antibiotics on growth rate and EC_{50} is the antibiotic concentration at which half this maximum effect is reached. Furthermore, $\Psi_{\min}$ is the growth rate at large antibiotic concentrations (which is often negative and thus a death rate) and zMIC is the 'pharmacodynamic MIC'. Both models are equivalent to our formulation, and after some algebra we arrive at

$$\alpha(B) = \Psi_{\max} \frac{1 - \left(\frac{B}{\mathrm{EC}_{50} / \left(\left(\frac{E_{\max} - \Psi_{\max}}{\Psi_{\max}} \right)^{1/\kappa} \right)} \right)^\kappa}{1 + \left(\frac{B}{\mathrm{EC}_{50} / \left(\left(\frac{E_{\max} - \Psi_{\max}}{\Psi_{\max}} \right)^{1/\kappa} \right)} \right)^\kappa / \left(\frac{E_{\max} - \Psi_{\max}}{\Psi_{\max}} \right)} \tag{S1.7a}$$

$$= \Psi_{\max} \frac{1 - (B/\mathrm{zMIC})^\kappa}{1 + (B/\mathrm{zMIC})^\kappa / \left(-\frac{\Psi_{\min}}{\Psi_{\max}} \right)} , \tag{S1.7b}$$

from which this equivalence can be easily discerned.

S1.1.1 Solutions to different decay mechanisms with constant death rate

At first, we treat the regime with a constant death-rate, $\alpha(B) \approx -\alpha_0\gamma$. In the ensuing calculation, we rescale time,

$$T \equiv \alpha_0 \gamma t , \tag{S1.8}$$

such that bacterial death rate is 1 in these dimensionless units. With this rescaling, the population dynamics is simply $\partial_T N = -N$. We immediately find the solution for the microbial population as $N(T) = N_0 \exp(-T)$. This gives the time $T_1 = \log N_0$ for survival in antibiotics: at this time, only one cell would remain, $(N(T_1) = 1)$, if antibiotics are still present, and the population is essentially extinct. The exponential decay of the population size allows to solve the dynamics of the antibiotic concentration

in other cases as well. In order to obtain these solutions for the antibiotic dynamics, we introduce its logarithmic concentration,

$$L \equiv \log\big(B/\mu\big) \ , \tag{S1.9}$$

which simplifies the dynamics. In all kinetic cases defined above the dynamics of antibiotics itself is linear in B, see Eqs. (S1.3). Thus, when we multiply each equation with $1/B$, we can use the identity $\frac{1}{B}\partial_t B = \alpha_0\gamma\,\partial_T L$, and the dynamics $\partial_T L$ is itself independent of L, which decouples the equations for N and L partially. The change of time units from t to T for the derivation introduces the coefficient $\alpha_0\gamma$. Using the logarithmic antibiotic concentration L, and inserting the solution $N(T) = N_0\exp(-T)$ for the population size, we can integrate all trajectories $L(T)$, and obtain

$$\text{Enzymatic degradation} \qquad L(T) = L_0 - \frac{\epsilon\rho}{(\alpha_0\gamma)^2}N_0\Big(e^{-T} - 1 + T\Big) \ , \tag{S1.10a}$$

$$\text{Absorption and Internal degradation} \qquad L(T) = L_0 - \frac{\zeta}{\alpha_0\gamma}N_0\Big(1 - e^{-T}\Big) \ , \tag{S1.10b}$$

$$\text{Binding to dead cell fragments} \qquad L(T) = L_0 - \frac{\nu}{\alpha_0\gamma}N_0\Big(e^{-T} - 1 + T\Big) \ , \tag{S1.10c}$$

$$\text{Spontaneous environmental degradation} \qquad L(T) = L_0 - \frac{\chi}{\alpha_0\gamma}T \ . \tag{S1.10d}$$

In these solutions, we observe that every parameters occurs in a single coefficient, and thus can be combined into an effective and dimensionless parameter. We define the timescale separation τ for each of the different degradation mechanics as,

$$\tau = \left\{ \frac{(\alpha_0\gamma)^2}{\epsilon\rho}, \ \frac{\alpha_0\gamma}{\zeta}, \ \frac{\alpha_0\gamma}{\nu}, \ \frac{\alpha_0\gamma}{\chi} \right\} \ , \tag{S1.11}$$

in the same order as above. Independent of the mechanism, τ is always the ration of the rate for bacterial death over the rate of antibiotic degradation. In the case of enzymatic degradation, which consists of two steps with enzyme production and then degradation, the death rate is squared to make τ dimensionless.

Now checking for population survival, we evaluate the antibiotic concentration $L(T_1)$ at time $T_1 = \log N_0$. If $L(T_1) < 0$ (which translates to $B(T_1) < \mu$), then the antibiotic concentration has been reduced far enough, that the population reached already positive values for its growth rate $\alpha(B)$, and started growing before it reached a single cell. Conversely, if still $L(T_1) > 0$, then the population would decay more and go extinct. Inserting $T_1 = \log N_0$ into each of the solutions in Eq. (S1.10), evaluating the condition $L(T_1) < 0$, and finally reverting back to B_0 from L_0 yields the expressions

$$\text{Enzymatic degradation} \qquad N_0(\log N_0 - 1) > -1 + \tau\log\big(B_0/\mu\big) \ , \tag{S1.12a}$$

$$\text{Absorption and Internal degradation} \qquad N_0 > 1 + \tau\log\big(B_0/\mu\big) \ , \tag{S1.12b}$$

$$\text{Binding to dead cell fragments} \qquad N_0(\log N_0 - 1) > -1 + \tau\log\big(B_0/\mu\big) \ , \tag{S1.12c}$$

$$\text{Spontaneous environmental degradation} \qquad \log N_0 > 1 + \tau\log\big(B_0/\mu\big) \ . \tag{S1.12d}$$

to antibiotic concentration B_0 instead of L_0. With the exception of spontaneous environmental degradation, we neglect the logarithmic population size dependence, and arrive at the universal expression for the MSI curve,

$$N_0 \gtrsim \tau\log\big(B_0/\mu\big) \ , \tag{S1.13}$$

where we neglected the additive constant $+1$ as population size N_0 is usually large enough that it does not matter. The main text also dismisses this constant, however, it is kept for all derivations in this supplemental material.

S1.1.2 Solutions by linear approximation close to MIC

Now we focus on the dynamics of competition when the initial antibiotic concentrations is intermediate, close to $B_0/\mu \approx 1$. In this regime, antibiotic-dependent growth rate is approximated with the linear expansion in (S1.5b):

$$\alpha(B) \approx -\alpha_0\lambda \log\big(B/\mu\big) \;. \tag{S1.14}$$

We utilize again a rescaling of time and the logarithmic antibiotic concentration

$$T \;\equiv\; \alpha_0\lambda t \;, \tag{S1.15a}$$
$$L \;\equiv\; \log\big(B/\mu\big) \;. \tag{S1.15b}$$

Then, the equation for population dynamics takes the form

$$\partial_T N = -LN. \tag{S1.16}$$

For each of the four reactions specified above, we define a dimensionless parameter τ, given by (in order of the reactions)

$$\tau = \left\{ \frac{(\alpha_0\lambda)^2}{\epsilon\rho}, \frac{\alpha_0\lambda}{2\zeta}, \frac{\alpha_0\lambda}{\nu}, \frac{\alpha_0\lambda}{2\chi} \right\} \;. \tag{S1.17}$$

All of these parameters are the ratios of timescales between population death and antibiotic degradation. We treat each mechanism is a separate section in the following.

S1.1.2.1 Enzymatic degradation

For enzymatic degradation, in the newly defined variables the equations are

$$\partial_T L = -\frac{\epsilon}{\alpha_0\lambda}E \;, \qquad \partial_T E = \frac{\rho}{\alpha_0\lambda}N \;. \tag{S1.18}$$

Differentiating the first equation again with respect to T, then using the second equation for $\partial_T E$, we find two coupled equations for population size and antibiotics

$$\partial_T N \;=\; -LN \;, \tag{S1.19a}$$
$$\partial_T^2 L \;=\; -\frac{1}{\tau}N \;. \tag{S1.19b}$$

We cannot state explicit closed form solutions for this case. Nevertheless, we can assume a constant exponential decay of the bacterial population, which is most of the time is close to the exact solution (checked numerically). Only close to the turning point (where bacteria start to grow again due to degraded antibiotics), we expect significant deviations from this simple exponential decay.

To this end, we use $N(T) \approx N_0 \exp\big(-L_0T\big)$, which can be integrated twice to obtain the trajectory $L(T)$. Initial conditions for the latter are $(\partial_T L)(0) = 0$ (no enzyme present at the beginning) and $L(0) = L_0$. The solutions are

$$N(T) \;\approx\; N_0 \exp\big(-L_0T\big) \;, \tag{S1.20a}$$
$$L(T) \;\approx\; L_0 - \frac{N_0}{\tau L_0^2}\Big(\exp\big(-L_0T\big) - 1 + L_0T\Big) \;. \tag{S1.20b}$$

Estimating the time $T_1 = (\log N_0)/L_0$ from inverting $N(T_1) = 1$, we compute the condition for survival of the bacterial population again from $L(T_1) < 0$. Algebraic rearrangements of this inequality lead to

$$N_0(\log N_0 - 1) > \tau L_0^3 - 1 \;, \tag{S1.21}$$

which in turn can be expressed in the Lambert-W function $\mathcal{W}$ (also called product-logarithm),

$$N_0 > \frac{\tau L_0^3 - 1}{\mathcal{W}\big((\tau L_0^3 - 1)/e\big)} \, . \tag{S1.22}$$

For large values of τL_0^3 (note that $\tau \sim \mathcal{O}(10^6)$, cf. Fig. 5), we have the series of approximations $X/\mathcal{W}(X) \approx X/\log X \approx X$. This logarithmic correction arising from $\mathcal{W}$ is usually small, and we neglect it to arrive at the MSI curve,

$$N_0 \gtrsim \tau L_0^3 = \tau \big(\log\big(B_0/\mu\big)\big)^3 \, . \tag{S1.23}$$

However, in contrast to before we find an exponent 3 for the (logarithmic) antibiotic concentration. In terms of our experiments, this would introduce a sharper bend on the plate close to the transition to the vertical part. For larger initial concentrations B_0, however, the MSI curve transitions into the solution with constant death rate treated before.

S1.1.2.2 Absorption and internal degradation

The dynamics for absorption and internal degradation can be solved analytically. This mechanism assumes a first order mass-action absorption reaction of antibiotics to cells, and then immediate degradation of antibiotics inside the cell (or at least very fast compared to absorption). The system of differential equations is given by

$$\partial_T N = -LN \, , \tag{S1.24a}$$
$$\partial_T L = -\frac{1}{2\tau} N \tag{S1.24b}$$

where τ is the second definition in (S1.17). Upon taking the ratio of both of these equations, we find $\frac{dN}{dL} = 2\tau L$. This auxiliary differential equation can be integrated to arrive at $N(L) = \tau L^2 + a$ with a an integration constant. Consequently, (S1.24b) becomes $\partial_T L = -L^2/2 - a/(2\tau)$. Although non-linear, this differential equation has a solution in trigonometric functions, and we have after inserting $L(T)$ into $N(L)$ the two solutions

$$L(T) = \sqrt{\frac{a}{\tau}} \tan\left[b - \sqrt{\frac{a}{\tau}}\frac{T}{2}\right] \, , \tag{S1.25a}$$
$$N(T) = a \cos^{-2}\left[b - \sqrt{\frac{a}{\tau}}\frac{T}{2}\right] \, , \tag{S1.25b}$$

where b is the second integration constant. Using the initial conditions $N(0) = N_0$ and $L(0) = L_0$, we find

$$L(T) = \sqrt{\frac{N_0}{\tau} - L_0^2} \tan\left[\arctan\left[\sqrt{\frac{\tau L_0^2}{N_0 - \tau L_0^2}}\right] - \sqrt{\frac{N_0}{\tau} - L_0^2}\frac{T}{2}\right] \, , \tag{S1.26a}$$
$$N(T) = \frac{N_0 - \tau L_0^2}{\cos^2\left[\arctan\left[\sqrt{\frac{\tau L_0^2}{N_0 - \tau L_0^2}}\right] - \sqrt{\frac{N_0}{\tau} - L_0^2}\frac{T}{2}\right]} \, . \tag{S1.26b}$$

Although these expressions look complicated, we can utilize the special trigonometric functions to proceed. The arguments of tan and $\cos^{-2}$ in these solutions are identical, and we know that the zeros of tan correspond to minima of $\cos^{-2}$ with value 1 $((\tan[X] = 0) \Leftrightarrow (\cos^{-2}[X] = 1), \; \forall X)$. Thus, we know that

the time T_L, defined as $L(T_L) = 0$ leads to $N(T_L) = N_0 - \tau L_0^2$, which we have to check if it is bigger or smaller than one. Consequently, we obtain the MSI curve

$$N_0 \gtrsim 1 + \tau L_0^2 = 1 + \tau\big(\log\big(B_0/\mu\big)\big)^2 \ , \tag{S1.27}$$

from rearranging this condition.

S1.1.2.3 Irreversible binding to cell fragments

Another degradation mechanism is irreversible binding of antibiotics to dead cell fragments and their consequent inactivation. The dynamic equations in this case are

$$\partial_T N = -LN \ , \tag{S1.28a}$$
$$\partial_T L = -\frac{1}{\tau}\big(N_0 - N\big) \ . \tag{S1.28b}$$

We approximate an exponential decay as solution to the bacterial population size, and then integrate the second equation for the (logarithmic) antibiotic concentration. This yields

$$N(T) \approx N_0 \exp\big(-L_0 T\big) \ , \tag{S1.29a}$$
$$L(T) \approx L_0 - \frac{1}{\tau L_0} N_0 \Big(\exp\big(-L_0 T\big) - 1 + L_0 T\Big) \ . \tag{S1.29b}$$

Using these solutions, we again check $L(T_1) < 0$, which translates into the condition on the minimal population size

$$N_0\big(\log N_0 - 1\big) > \tau\big(\log\big(B_0/\mu\big)\big)^2 \ . \tag{S1.30}$$

S1.1.2.4 Spontaneous environmental degradation

As the simplest of all cases, we treat spontaneous environmental degradation. The set of two equations is given by

$$\partial_T N = -LN \ , \tag{S1.31a}$$
$$\partial_T L = -1/(2\tau) \ . \tag{S1.31b}$$

This system can be solved almost trivially: We immediately find $L(T) = L_0 - T/(2\tau)$, which we can insert into the dynamics of N to find $N(T) = N_0 \exp\big(T^2/(4\tau) - L_0 T\big)$. This solution for the population size has a minimum at time $T_{\min} = 2\tau L_0$. Thus, the population will survive if $N(T_{\min}) = N_0 \exp\big(-\tau L_0^2\big) > 1$. Reverting back to original variables yields the condition

$$\log N_0 > \tau L_0^2 = \tau\big(\log\big(B_0/\mu\big)\big)^2 \ . \tag{S1.32}$$

Here, we find a logarithmic dependence on initial population size N_0, which deviates from the MSI curve.

S1.1.3 Effects of exponent

Overall, section S1.1.2 showed that for initial antibiotic concentrations close to μ (and $\alpha(B) \approx -\alpha_0 \lambda \log\big(B/\mu\big)$), the exponent ξ in the MSI curve increases by 1 compared to the same antibiotic degradation mechanism with a constant death rate ($\alpha(B) \approx -\gamma\alpha_0$, section S1.1.1). This discrepancy arises because in

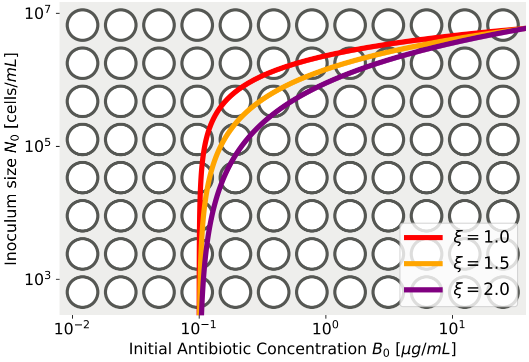

Figure S1.2: **Effects of exponent ξ on MSI curve.** For all three MSI curves, μ_{eff} is identical, and τ is chosen such that the curves intersect he plate edge at the same point.

our solutions the population decay in the initial phase of the dynamics is $N(t) \approx N_0 \exp(-L_0 T)$ vs. $N(T) \approx N_0 \exp(-T)$. This additional factor L_0 in the rate leads to an factor $1/L_0$ in the time T_1, which in turn appears in the MSI inequality, and increases the exponent of L_0 by 1. This larger exponent leads to a slightly different shape of the MSI, where the bending of the curve is not as sharp as in the step-function regime treated in section S1.1.1 (see Fig. S1.2). However, for the dilution series used in our experimental setup, the range with a valid linear approximation for the growth rate, Eq. (S1.14), is likely small. Thus, we use the the first approximation for our fitting algorithm throughout and neglect this change in the exponent when estimating μ_{eff} and τ from our experiments.

S1.2 Privatization effects of an extracellular enzyme

The main text only contained an overview of the steps involved in deriving a model that includes dynamical privatization. Here, we present a more detailed account of these computations.

Our original model included bacterial growth, together with production of an enzyme (eg. β-lactamase) that reduces antibiotics. Now, we include additional ODEs explicitly describing the time-evolution inside and outside of cells,

$$\begin{aligned}
\partial_t N &= \alpha(B_{\text{in}})\, N && \text{(S1.33a)}\\
\partial_t E_{\text{in}} &= \rho + \sigma_{\text{E}}(E_{\text{out}} - E_{\text{in}}) && \text{(S1.33b)}\\
\partial_t E_{\text{out}} &= \sigma_{\text{E}} \eta N \big(E_{\text{in}} - E_{\text{out}}\big) && \text{(S1.33c)}\\
\partial_t B_{\text{in}} &= -\epsilon E_{\text{in}} B_{\text{in}} + \sigma_{\text{B}} \big(B_{\text{out}} - B_{\text{in}}\big) && \text{(S1.33d)}\\
\partial_t B_{\text{out}} &= -\epsilon E_{\text{out}} B_{\text{out}} + \sigma_{\text{B}} \eta N \big(B_{\text{in}} - B_{\text{out}}\big)\ . && \text{(S1.33e)}
\end{aligned}$$

Enzyme E and antibiotics B are transported between the inside and the outside with rates σ_{E} and σ_{B}, respectively. The volume-separation factor η indicates how much slower the concentrations outside cells change, compared to the rather fast dynamics within. This factor occurs, as the volume of the culture medium V_{medium} is usually much larger as the volume of all cells V_{cells}, and we have to account for the fact that a single molecule passing through the membrane affects concentrations in different ways for internal and external concentrations. Thus, we need to couple the transport processes via a factor

$V_{\mathrm{cells}}/V_{\mathrm{medium}} = \eta N$, where η is the ratio of volumes for a single cell. As long as $\eta \ll 1$, we can assume that adjustment of inner concentrations (E_{in} and B_{in}) is essentially instantaneous compared to the dynamics outside cells. Explicitly, for our experiments we have $V_{\mathrm{medium}} = 2 \cdot 10^{-4} L$ and $V_{\mathrm{cell}} \approx 10^{-15} L$, such that $\eta \approx 5 \cdot 10^{-12}$. Hence, we can make an approximation that these internal concentrations are stationary, and set both $\partial_t E_{\mathrm{in}} \approx 0$ and $\partial_t B_{\mathrm{in}} \approx 0$. This allows to algebraically rearrange Eqs. (S1.33b) and (S1.33d) to express inner concentrations as functions of the (slower varying) outer concentrations,

$$E_{\mathrm{in}} \approx E_{\mathrm{out}} + \frac{\rho}{\sigma_{\mathrm{E}}} \,, \tag{S1.34a}$$

$$B_{\mathrm{in}} \approx \left(1 + \frac{\epsilon}{\sigma_{\mathrm{B}}}\left(E_{\mathrm{out}} + \frac{\rho}{\sigma_{\mathrm{E}}}\right)\right)^{-1} B_{\mathrm{out}} \,. \tag{S1.34b}$$

These relations indicate that the effective enzyme concentration inside cells is higher by an additive term ρ/σ_{E} compared to outside cells. Moreover, assuming the outside enzyme concentration remains small relative to inside, $E_{\mathrm{out}} \ll \rho/\sigma_{\mathrm{E}}$ the effective antibiotic concentration is reduced by a factor $\big(1 + \epsilon\rho/\sigma_{\mathrm{E}}\sigma_{\mathrm{B}}\big)$.

Inserting these two expressions in the outside dynamics leads to

$$\partial_t E_{\mathrm{out}} \approx \eta\sigma_{\mathrm{E}} N\Big(\big(E_{\mathrm{out}} + \frac{\rho}{\sigma_{\mathrm{E}}}\big) - E_{\mathrm{out}}\Big) = \eta\rho N \,, \tag{S1.35a}$$

$$\partial_t B_{\mathrm{out}} \approx -\epsilon E_{\mathrm{out}} B_{\mathrm{out}} - \sigma_{\mathrm{B}}\eta N \left(\frac{\frac{\epsilon}{\sigma_{\mathrm{B}}}\left(E_{\mathrm{out}} + \frac{\rho}{\sigma_{\mathrm{E}}}\right)}{1 + \frac{\epsilon}{\sigma_{\mathrm{B}}}\left(E_{\mathrm{out}} + \frac{\rho}{\sigma_{\mathrm{E}}}\right)}\right) B_{\mathrm{out}} \,. \tag{S1.35b}$$

In the first line, we recover the expected dynamics of enzyme production, $\partial_t E = \rho\eta N$, where the original production rate ρ needs to be adjusted to also incorporate the volume-separation factor η. The dynamics of antibiotics in Eq. (S1.35b) now exhibits two terms, where the first term can be attributed to degradation outside the cells, while the second term indicates degradation inside cells. Numerical analysis of the contributions for both to the overall decay of antibiotics shows that external degradation is usually (much) larger. Thus, we will continue to use the results of the MSI curve from the (simpler) model without explicit internal and external concentrations, as derived in the previous sections.

These considerations change the timescale separation τ for the explicit treatment of internal concentrations to

$$\tau \approx \frac{(\alpha_0\gamma)^2}{\epsilon\rho\eta} \,, \tag{S1.36}$$

which contains an additional factor η. Note, however, that this does not change the value of τ itself, but rather which values we have to attribute to the microscopic parameters ρ (and maybe ϵ) when conducting simulations that are supposed to match either experiments or simulations for the dynamics without these explicit inner concentrations.

So far, we always measured antibiotic concentrations B in units of multiples of μ. The dynamics of B itself, see Eqs. (S1.33d) and (S1.33e), is invariant under multiplication with a scale factor, that can be chosen arbitrarily (and we used μ to fulfill this role). The only point in our model, where we need proper units for the antibiotic concentrations, is to quantify the effects on growth rate, defined via Eq. (S1.5a), where the ratio B/μ enters. Moreover, in the derivation of privatization effects above, we found that the cell effectively sees a slightly reduced concentration of antibiotics, B_{in}, as can be measured in media, B_{out}. Thus, we can write

$$L = \log\big(B_{\mathrm{in}}/\mu\big) \approx \log\left(\frac{B_{\mathrm{out}}}{(1 + \epsilon\rho/\sigma_{\mathrm{B}}\sigma_{\mathrm{E}})\mu}\right) = \log\big(B_{\mathrm{out}}/\mu_{\mathrm{eff}}\big) \,. \tag{S1.37}$$

This allows to state the overall dynamics in the outer concentration B_{out} as we did in Eq. (S1.37), we can now relate the effective single cell MIC μ_{eff} to the underlying dynamical parameters. In the main text we introduced the privatization parameter $\Phi = (\epsilon\rho)/(\sigma_{\text{B}}\sigma_{\text{E}})$ that occurs here. Writing Φ in terms of the timescale separation τ, Eq. (S1.36), we obtain a parameter φ characterizing the privatization effect,

$$\varphi = \frac{(\alpha_0\gamma)^2}{\eta\sigma_{\text{E}}\,\sigma_{\text{B}}}\ , \tag{S1.38}$$

such that $\Phi = \varphi/\tau$ holds.

Specifically, we find that the antibiotic concentration where population growth turns into death is given by

$$\mu_{\text{eff}} = \left(1 + \frac{\varphi}{\tau}\right)\mu\ . \tag{S1.39}$$

This relation (S1.39) is linear in $1/\tau$, which is the singular parameter characterizing an allele. In general, we expect φ to be a (different) constant for both our sets of experiments with *E. coli* MG1655 (Fig. 5A) and *E. coli* BW27783 (Fig. 5B), because only changing only the TEM-1 allele should not affect either growth/death processes of the cell, and neither its permeability for antibiotics and/or enzyme.

Susceptible cells should correspond to a limit of $1/\tau \to 0$, as then either production is negligible ($\rho \to 0$) or the enzyme is not effective ($\epsilon \to 0$). As a sanity check, extrapolating the μ_{eff} values to $1/\tau = 0$ should give the μ value measured in kill curve experiments.

S1.3 Parameter estimation on plates

The experimental protocol how to generate the dilution series on 96-well plates was already described in the main text in the Material and Methods section. Here we provide the steps, including additional technical details, for extracting τ and μ_{eff} from these plates.

S1.3.1 Otsu's Method for threshold detection

The first step in extracting the parameters is to estimate the threshold between growth and no-growth in an automated way. We use a slightly modified version of Otsu's method [3] for finding this threshold. Originally, this method has been proposed for binarizing images into black and white by separating the all observed values into two classes. It works by minimizing variance within a class, or equivalently, by maximizing variance between the two classes.

In order to compute the threshold, we first sort the OD values from all plates of all experiments into a vector $\mathbf{a}$ of length $M \cdot R$ (with $M = 96$ the number of OD values on one plate and R the number of all plates),

$$\mathbf{a} \equiv \text{sort}\left(\log \mathbf{OD}\right)\ . \tag{S1.40}$$

Here, we use logarithmic values, as usually only the magnitude of the growth and no-growth OD values is important, which allows to get a clearer threshold value. We slightly modify Otsu's method to use non-equally spaced bins with each a single observation instead of equally spaced bins with (potentially) multiple observations. Then, the computation can be reduced to finding the index m that maximises the expression,

$$m = \underset{1<m<MR}{\arg\max}\left(\frac{\left(m\sum_{i=1}^{MR} a_i - MR\sum_{j=1}^{m} a_j\right)^2}{m(MR-m)}\right)\ . \tag{S1.41}$$

The threshold between growth and no-growth is then defined as intermediate between two consecutive points in $\mathbf{a}$, $G = \beta a_m + (1-\beta) a_{m+1}$ with $\beta = 1/2$. All OD values above this threshold G are considered growing, while all values below are considered no-growth.

S1.3.2 Parameter estimation

With the set of points for the threshold between growth and no-growth, obtained in the previous step, we first transform both axes to their logarithmic scales, which gives a set of the initial conditions $\big(\log(N-1)\big)_i$, $\big(\log B\big)_i$ for each of the wells. Then we fit the (non-linear) functional form

$$\big(\log B\big)_i = \frac{1}{\tau} \exp\Big(\big(\log(N-1)\big)_i\Big) + \log \mu_{\mathrm{eff}} \ , \tag{S1.42}$$

to first obtain values for $(1/\tau)$ and intercept $(\log \mu_{\mathrm{eff}})$ using Python's *scipy.optimize.curve_fit*. Then, using the Python module *uncertainties*, we use the covariance matrix between these two fitted parameters to compute the actual values τ and μ_{eff}, in addition to their standard deviations, which are shown in Fig. 5. The advantage of using logarithmic values for both axes of initial population size and antibiotic concentration is that distances are weighted according to distances on the plate. Evaluating the $\exp(\log N_i)$ term to obtain the direct linear dependency on N would emphasize large initial populations too much in the fitting procedure.

S1.4 Notation

Symbol	Description	Units	Comment
N	Population Size	[cells/mL]	
B	Antibiotic Concentration	[$\mu g/mL$]	
E	Enzyme Concentration	[$\mu g/mL$]	Enzyme is β-Lactamase
B_{in}	Internal Antibiotic Conc.	[$\mu g/mL$]	Used in extended model
B_{out}	Outer Antibiotic Conc.	[$\mu g/mL$]	Used in extended model
E_{in}	Internal Enzymatic Conc.	[$\mu g/mL$]	Used in extended model
E_{out}	Outer Enzymatic Conc.	[$\mu g/mL$]	Used in extended model
L	Logarithmic Antiobiotic Conc.	1	$L = \log(B/\mu)$
$\alpha(B)$	Effective Growth-/Death-Rate	[$1/h$]	Net rate for population
α_0	Growth Rate without antibiotics	[$1/h$]	
μ	(Pharmacodynamic) MIC	[$\mu g/mL$]	At $B = \mu$ growth switches to death
γ	Maximal Death Rate multiplier	1	$\alpha(B = \infty) = -\gamma\alpha_0$
κ	Steepness	1	Steepness in switching between growth and death
λ		1	Abbreviation for $\lambda = \kappa\gamma/(1+\gamma)$
N_0	Inoculum Size	[cells/mL]	$N_0 = N(t=0)$
B_0	Initial Antibiotic Concentration	[$\mu g/mL$]	$B_0 = B_{\text{out}}(t=0)$
μ_{eff}	Effective MIC	[$\mu g/mL$]	MIC from extended model
τ	Timescale Separation	[cells/mL]	Ratio of death rate to degradation rate, exact definition depends on model. See Eqs. (S1.11) and (S1.17).
ξ	Exponent in MSI	1	Usually close to 1
ϵ	Catalytic Efficiency	[$mL/(\mu g\ h)$]	
ρ	Enzyme Production Rate	[$\mu g/$(cell h)]	
ρ	Enzyme Production Rate	[$\mu g/(mL\ h)$]	Different units in extended model
σ_{B}	Permeability for Antibiotics	[$1/h$]	
σ_{E}	Permeability for Enzyme	[$1/h$]	
φ	Permeability Parameter	[mL/cell]	Ratio of death rate to permeabilities
Φ	Privatization Parameter	[1]	$\Phi = \varphi/\tau$
η	Volume Separation	[mL/cell]	Used in extended model
ζ	Degradation Rate	[$mL/$(cell h)]	Additional models
ν	Adsorption Rate	[$mL/$(cell h)]	Additional models
χ	Degradation Rate	[$1/h$]	Additional models

Table S1.1: **Notation used in this manuscript.**

S1.5 Additional Plots

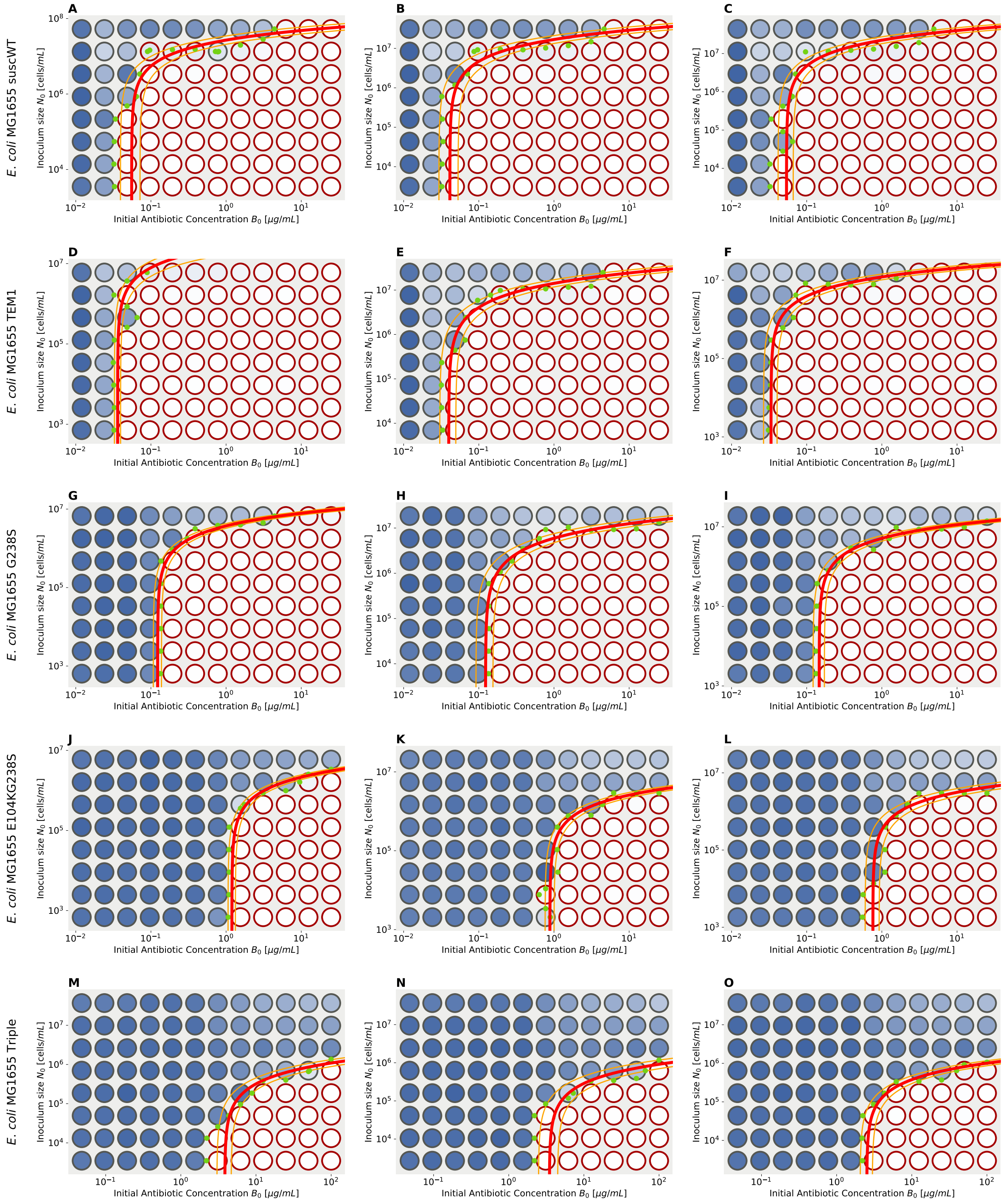

Figure S1.3: **MSI curve fits for *E. coli* MG1655.** Obtained μ_{eff} and τ are shown in Fig. 5A. All 5 alleles of TEM-1 (top - bottom: no TEM-1, TEM1, G238S, E104K-G238S, Triple) with 3 replicates each (horizontal) are shown. Catalytic efficiency ϵ increases from from top to bottom. Green dots indicate estimated transition points between growth and no-growth, as described in section S1.3.1.

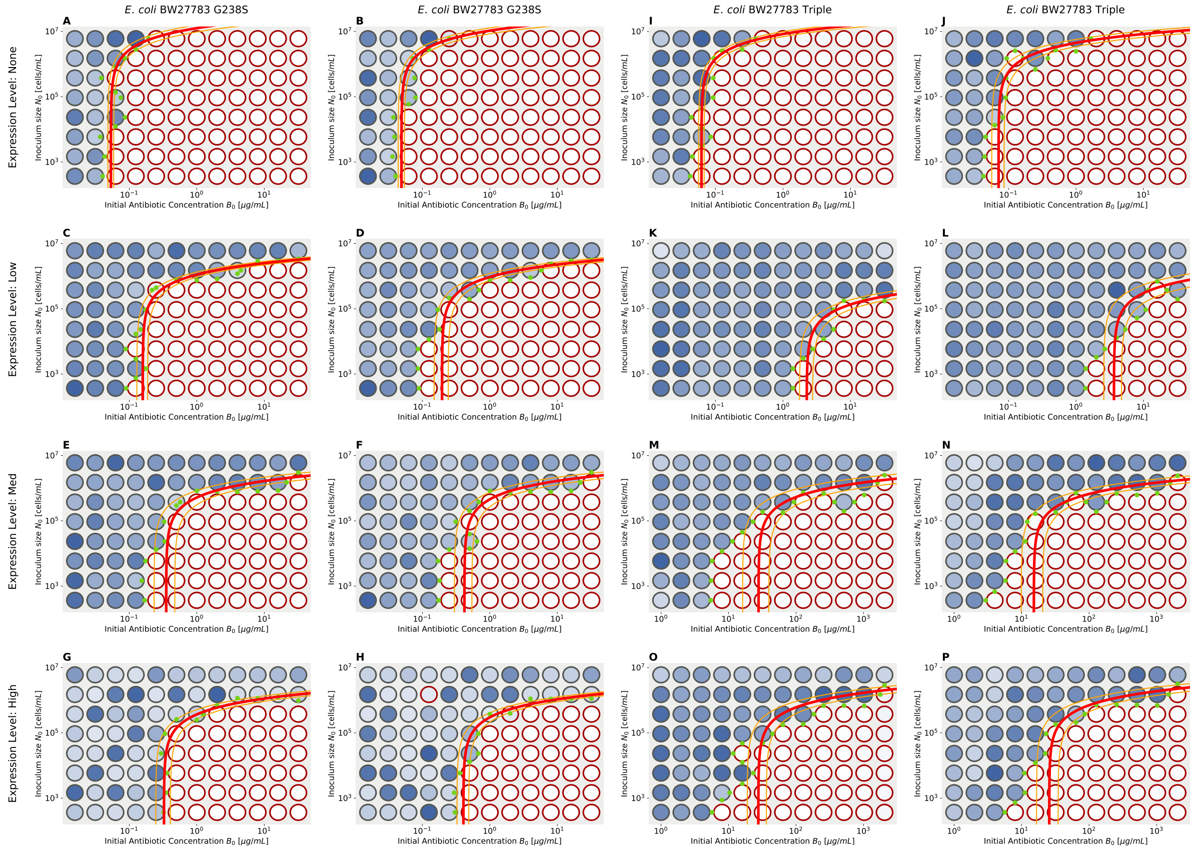

Figure S1.4: **MSI curve fits for *E. coli* BW27783.** Obtained μ_{eff} and τ are shown in Fig. 5B. All 20 plates for two variants of the TEM-1 allele (G239S mutation in panels A-H, triple mutation in panels I-P) that exhibited four different expression levels ρ, increasing from top (None/Leaky expression) to bottom (High expression) rows. Green dots indicate estimated transition points between growth and no-growth, as described in section S1.3.1.

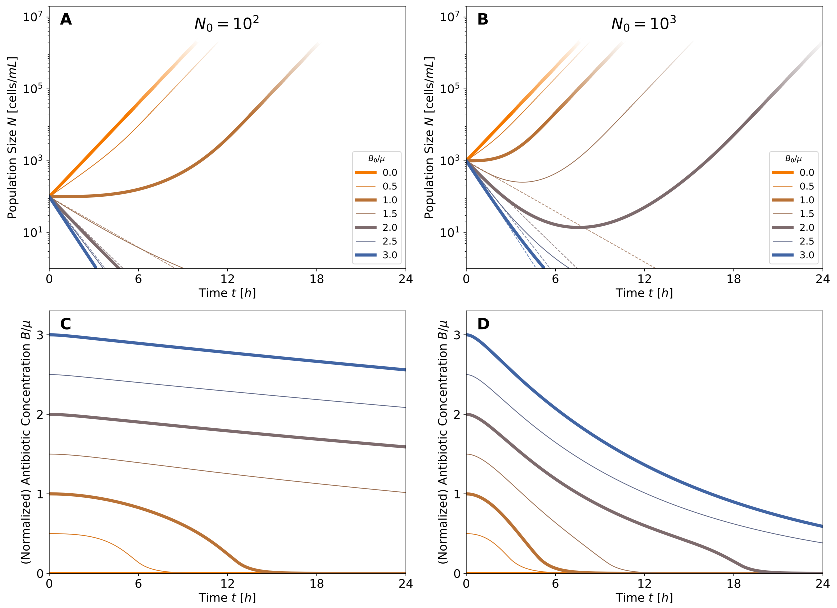

Figure S1.5: **Trajectories of Population Size and (Normalized) Antibiotic Concentration.** Extension of the data presented in Fig. 2 of the main text with panels C and D, which show the associated (normalized) antibiotic concentrations B/μ. In addition, we also show the exponential approximation $N(t) = N_0 \exp(-\lambda L_0 t)$ used in our derivations when $B_0/\mu > 1$ as dashed lines in panels A and B, which are close to the exact numerical solution as long as the population is decaying.

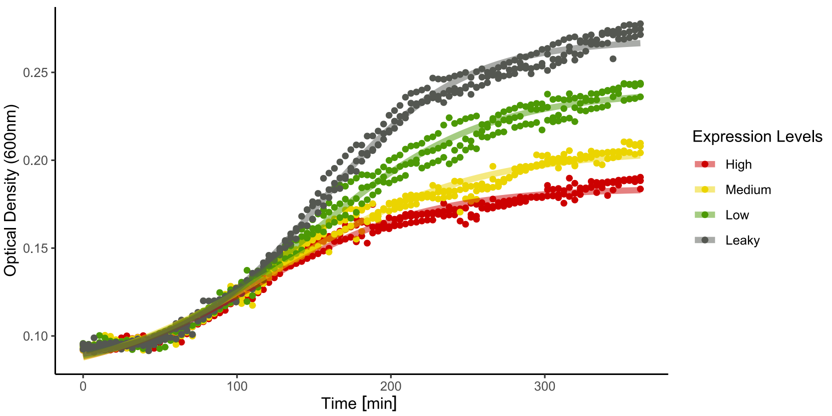

Figure S1.6: Growth curves of the four different expression levels of BW27783 expressing TEM-1 from the pBAD322T vector.

References

[1] Tatiana Artemova, Ylaine Gerardin, Carmel Dudley, Nicole M Vega, and Jeff Gore. Isolated cell behavior drives the evolution of antibiotic resistance. *Molecular systems biology*, 11(7):822, 2015. doi: 10.15252/msb.20145888.

[2] Roland R Regoes, Camilla Wiuff, Renata M Zappala, Kim N Garner, Fernando Baquero, and Bruce R Levin. Pharmacodynamic functions: a multiparameter approach to the design of antibiotic treatment regimens. *Antimicrobial agents and chemotherapy*, 48(10):3670–3676, 2004. doi: 10.1128/AAC.48.10.3670-3676.2004.

[3] Nobuyuki Otsu. A threshold selection method from gray-level histograms. *IEEE transactions on systems, man, and cybernetics*, 9(1):62–66, 1979. doi: 10.1109/TSMC.1979.4310076.
